# tDCS-induced alterations in GABA concentration within primary motor cortex predict motor learning and motor memory: A 7 T magnetic resonance spectroscopy study

**DOI:** 10.1016/j.neuroimage.2014.05.070

**Published:** 2014-10-01

**Authors:** Soyoung Kim, Mary C. Stephenson, Peter G. Morris, Stephen R. Jackson

**Affiliations:** a Brain and Body Centre, School of Psychology, University of Nottingham, UK; b Sir Peter Mansfield Magnetic Resonance Centre, University of Nottingham, UK

**Keywords:** BOLD, blood-oxygen-level-dependent, fMRI, functional magnetic resonance imaging, GABA, γ-amino-butyric acid, M1, primary motor cortex, MRI, magnetic resonance imaging, MRS, magnetic resonance spectroscopy, NAA, N-acetylaspartate, NAAG, N-acetylaspartylglutamate, tDCS, transcranial direct current stimulation, TMS, transcranial magnetic stimulation, V1, primary visual cortex, Motor learning, Force adaptation, Magnetic resonance spectroscopy, tDCS, GABA

## Abstract

Transcranial direct current stimulation (tDCS) is a non-invasive brain stimulation technique that alters cortical excitability in a polarity specific manner and has been shown to influence learning and memory. tDCS may have both on-line and after-effects on learning and memory, and the latter are thought to be based upon tDCS-induced alterations in neurochemistry and synaptic function. We used ultra-high-field (7 T) magnetic resonance spectroscopy (MRS), together with a robotic force adaptation and de-adaptation task, to investigate whether tDCS-induced alterations in GABA and Glutamate within motor cortex predict motor learning and memory. Note that adaptation to a robot-induced force field has long been considered to be a form of model-based learning that is closely associated with the computation and ‘supervised’ learning of internal ‘forward’ models within the cerebellum. Importantly, previous studies have shown that *on-line* tDCS to the cerebellum, but not to motor cortex, enhances model-based motor learning. Here we demonstrate that anodal tDCS delivered to the hand area of the left primary motor cortex induces a significant reduction in GABA concentration. This effect was specific to GABA, localised to the left motor cortex, and was *polarity specific* insofar as it was not observed following either cathodal or sham stimulation. Importantly, we show that the magnitude of tDCS-induced alterations in GABA concentration within motor cortex predicts individual differences in both motor learning and motor memory on the robotic force adaptation and de-adaptation task.

## Highlights

• Ultra-high-field (7 T) magnetic resonance spectroscopy study of the effects of tDCS.
• Anodal tDCS leads to a polarity and site specific reduction in MRS-GABA.
• tDCS-induced changes in MRS-GABA in M1 predict model-based motor learning/memory.

## Introduction

The effects of transcranial direct current stimulation (tDCS) – in particular the modulation of cortical excitability – have most often been studied using transcranial magnetic stimulation (TMS) techniques. These studies have shown that the magnitude of the motor evoked potential induced by a single TMS pulse can be increased after anodal tDCS and decreased after cathodal tDCS (left M1-contralateral forehead montage) (Nitsche and Paulus, 2000, Nitsche et al., 2005), can outlast the stimulation duration by up to 90 min following a 1 mA stimulation (Nitsche and Paulus, 2001), and are dependent on synaptic activity changes. Pharmacological studies indicate that the cortical excitability changes induced by anodal tDCS and cathodal tDCS are both NMDA receptor-dependent (Liebetanz et al., 2002, Nitsche et al., 2003), whereas the changes induced by anodal tDCS are particularly dependent on GABA_A_ receptors (Nitsche et al., 2004). Stagg et al. (2009) demonstrated that anodal tDCS delivered to the left primary motor cortex (M1) led to a reduction in GABA concentration while cathodal tDCS to left M1 led to a reduction in both Glutamate and GABA concentration. The importance of GABA for motor learning has previously been shown in both animal (Hardwick et al., 2013, Trepel and Racine, 2000) and human studies (Ziemann et al., 1998, Ziemann et al., 2001) which demonstrate that GABA modulation plays a critical role in LTP-like plasticity within the motor cortex.

Importantly, a distinction has often been drawn between learning mechanisms involved in action selection and those involved in action execution. The former have been described as the formation of habitual responses, in which actions with a successful outcome are reinforced and are more likely to be repeated in the future (Graybiel, 2008). The basal ganglia nuclei and the midbrain dopamine projections are believed to play a key role in this form of learning and temporal-difference models have proposed that dopamine firing rates code for the reward prediction errors that are proposed as the training signal for reinforcement learning (Barto, 1995, Schultz et al., 1997) This form of learning is often referred to as *model-free* learning in that it is assumed to involve learning to estimate or predict the likely outcome (value or reward) of a given action (state) given an appropriate action-selection policy but does not make reference to an internal model of the state transitions associated with a given task. By contrast, *model-based* learning has been associated with goal-directed action in which the efficacies of candidate actions are evaluated with reference to an internal model of the task or state space. A key concept associated with this type of learning is that the selection of an appropriate action may involve a ‘mental simulation’ of potential outcomes (Dayan and Niv, 2008). Recent approaches to understanding how we learn to control our movements have proposed that fast and efficient motor behaviour relies upon predictive mechanisms that provide accurate estimates, or ‘internal models’, of the changing state of our body and the objects with which we interact. Internal ‘forward’ models are thought to compute dynamic estimates of the body state and to predict the sensory consequences of actions. Any discrepancies between the predicted and observed parameters of an action are used as training signals within a ‘supervised learning’ mechanism to increase or maintain the accuracy of the forward model (Wolpert and Ghahramani, 2000). It has been argued that the cerebellum may play a key role in the computation and supervised learning of internal ‘forward’ models of the motor apparatus (Wolpert et al., 1998).

In summary, model-free and model-based learning are thought to involve different learning mechanisms and brain systems (i.e., reinforced habit learning involving basal ganglia circuits and internal model formation within the cerebellum). While MRS has been used previously to study how tDCS-induced alterations in brain chemistry may predict model-free habit learning (Stagg et al., 2011) it has not to our knowledge been used to investigate how tDCS-induced plasticity influences model-based learning.

Galea et al. (2010) showed that anodal tDCS delivered to the cerebellum during learning (i.e., *online* tDCS) led to faster adaptation of reaching movements to a visuomotor transformation, whereas anodal tDCS applied to M1 during learning produced an increase in the retention of the learnt visuomotor transformation. This finding is consistent with an earlier finding reported by Reis et al. (2009) which demonstrated that anodal tDCS delivered to the primary motor cortex (M1) during motor skill learning (a variable force production task) led to a selective enhancement of *offline* tDCS after-effects (i.e., the post-stimulation consolidation of learning) relative to sham stimulation, but had no significant *online* effect on motor learning. Together these findings suggest that M1 may play a critical role in the consolidation of motor memory, but the effects of tDCS to M1 on motor learning remain unclear.

In the current study we investigated directly the extent to which tDCS-induced plasticity in M1, i.e., the after-effects of tDCS, and in particular any alterations in GABA and Glutamate concentration that may follow tDCS, might influence motor learning and motor memory using a robotic force adaptation and de-adaptation task. Importantly, the force adaptation task used in the current study is conceptually similar to the visuomotor adaptation task reported by Galea et al. (2010). Individuals were first required to adapt to a counter-clockwise force perturbation delivered by a robotic manipulandum during the execution of rapid aiming movements and, once learning had taken place, to de-adapt to the removal of the force perturbation.

Non-invasive investigation of GABA and Glutamate concentrations in-vivo is possible using MRS but it is challenging at conventional field strengths where these metabolites can only be measured using specialised edited MR sequences (Puts and Edden, 2012, Stagg, 2014). However many of the challenges in measuring GABA in-vivo using ^1^H MRS can be overcome by imaging at ultra-high-field strengths. For this reason in the current study we used ultra-high-field (7 T) MRS to measure the concentration of Glutamate, Glatamine, and GABA. Spectroscopy data were collected from the stimulation area within the left M1, from a mirror symmetrical region within the right M1, and from a control site located within the occipital cortex (primary visual cortex, V1).

## Materials and methods

### Participants

35 healthy participants (15 females, mean age: 21.1 ± 2.8 years, range: 18–29 years) were recruited from the University of Nottingham. All participants were right handed as assessed by the Edinburgh handedness inventory (Oldfield, 1971). None had a history of neurological disease or specific concerns over receiving tDCS and TMS or of being scanned within the MRI scanner. The study had received ethics approval from an appropriate local research ethics committee. Written informed consent was obtained from each participant, who were randomly assigned to either the anodal, cathodal, or sham tDCS conditions.

### Procedure

**Fig. 1:**
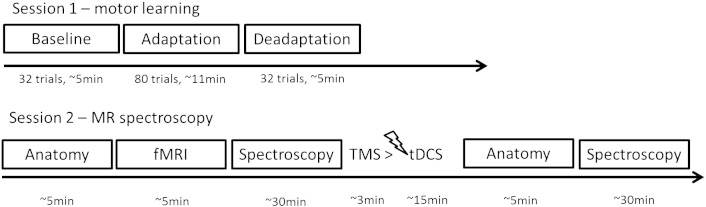
A graphical representation of the procedure.

Motor learning performance was measured using a force adaptation and de-adaptation task implemented on a vBot-2D robot. Neurochemical changes induced by tDCS were measured using ^1^H MR spectroscopy on a separate day (Fig. 1). For the MR spectroscopy session, data were collected twice (pre- and post-tDC stimulation). Participants received tDCS outside of the MR scanner between the two MRS sessions. The motor learning session was always conducted before MR spectroscopy session. There was in each case approximately one week between the motor learning and MRS sessions.

### Force adaptation task

The participants performed the force adaptation task using a two-dimensional planar robotic manipulandum (vBOT) that was designed for investigating dynamic motor control of the human arm (Howard et al., 2009). Participants could move the handle of the robot manipulandum freely in the horizontal plane and their movements were sampled at 1000 Hz. Similarly, forces could be imposed on the vBOT handle and these forces were also updated at a rate of 1000 Hz.

A projector and a semi-transparent screen were positioned horizontally 450 mm above the movement plane to show a cursor (white circle, radius: 7 mm) that represented the location of the handle, and a target (red circle, radius: 7 mm). Participants could see these images through a mirror that was positioned horizontally 220 mm above the movement plane (between the handle and the projector screen). This setting allowed the visual representation of the participants' movements to appear in the same spatial plane with their actual movement, and provided an intuitive experimental setting that did not require additional sensorimotor transformations (Preston et al., 2010).

**Fig. 2:**
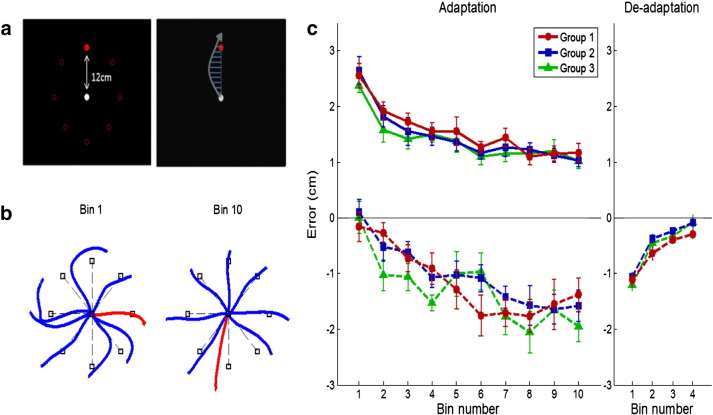
a. A graphical representation of the display used within the force adaptation task (left) and an illustration of the measurement of the perpendicular error from a straight line. b. Example trajectory of the first bin and the last bin (bin size = 8 trials, blue: force trials; red: catch trials). Note that, whereas force trials decrease in error with practice, errors during catch trials increase. c. Binned results of the force adaptation task of the adaptation phase (top: force trials; bottom: catch trials) and the de-adaptation phase for each group (solid line: force field; dashed line: null field). Groups 1, 2, and 3 each subsequently went on to receive anodal, cathodal, or sham tDCS in the following MRS session.

Participants were seated throughout and held the handle of the vBot during the task. Each trial started with an auditory warning that was followed immediately by the presentation of a target stimulus that could appear at one of eight radially arrayed positions (i.e. 45°, 90°, … 360°), each 12 cm from the central starting position (see Fig. 2a). The location of the target was pseudo-randomised such that, within each set of eight consecutive trials, each target location was presented only once. Participants were instructed to perform the task using rapid aiming movements towards the target. In particular, they were required to execute their movements so as to pass through the target position rather than stop at the target. Hand movement trajectories were recorded for a period of 3 s from the onset of each trial, after which the robot automatically returned the handle back to the central starting position. The perpendicular movement error was measured at 10 points that were equally distant (i.e., 10%, 20%, 30%, etc.) from the starting point to the target (see Fig. 2b). Negative error values indicate that the error was made in the direction opposite to the external force created by the robot. Each new trial commenced one second after the handle had returned to the starting position. If the peak velocity of the movement was greater than 80 cm/s or slower than 50 cm/s, a warning message of “too fast” or “too slow” was shown on the screen at the end of each trial.

The force adaptation task was divided into three phases: a baseline phase (32 trials); an adaptation phase (80 trials); and a de-adaptation phase (32 trials) [see Fig. 2c]. In the baseline and de-adaptation phases participants performed the task in a null force field and an external force was not applied during reaching movements. By contrast, during the adaptation phase, a velocity-dependent counter-clockwise (CCW) force field (20 N/s/m) was applied during reaching movements on the majority of trials. This force was not applied on a small proportion of catch trials that occurred pseudo-randomly within the adaptation phase. A catch trial was presented once randomly with each set of eight trials. During baseline and adaptation trials participants could view the cursor throughout their reaching movement. In the de-adaptation phase the cursor was not displayed during reaching so as to prevent the adaptation aftereffect disappearing too fast.

### Transcranial direct current stimulation

Participants were randomly assigned to three groups. Each group received anodal, cathodal, or sham stimulation to left hemisphere M1 region. Current was delivered to the scalp using a DC-STIMULATOR (neuroConn©) through two sponge-covered rubber electrodes (5 × 7 cm) soaked in saline solution. According to the polarity of the assigned group, the anodal or cathodal electrode was placed over the hand area of left M1 which had been identified for each participant using single pulse TMS. The other electrode was placed on the right supra-orbital area (Nitsche and Paulus, 2000). Anodal or cathodal tDCS was delivered with an intensity of 1.5 mA (current density: 0.043 mA/cm^2^) for 15 min. It was delivered in a single mode (continuous stimulation) with 8 s fade in and fade out (i.e., change from 0 to 1.5 mA, and vice versa). For the sham stimulation the same electrodes were placed at the same location for 15 min, but electrical current was delivered for only 15 s at the beginning of the stimulation period.

### fMRI BOLD and MR spectroscopy data acquisition

Magnetic resonance imaging data were acquired using a Philips Achieva 7 T magnetic resonance imaging scanner with a 32-channel SENSE radio-frequency head coil. A magnetisation prepared rapid gradient echo (MP-RAGE) anatomical image of the whole brain (120 slices, voxel size = 1 × 1 × 1 mm, TR = 7.3 ms) was obtained to aid the placement of three VOIs for spectroscopy. In order to localise the left and right M1 region, a functional MRI scan was carried out (EPI: TE/TR = 25/2000 ms, voxel size = 2 × 2 × 2 mm, field of view = 112 × 112 voxels, 30 slices) for on-line analysis. Participants wore prism glasses in the scanner allowing them to see a screen outside the scanner. The word LEFT or RIGHT was shown for 8 s after which the word REST was displayed for 32 s (8 trials in total). Participants were asked to perform a self-paced hand clenching task while LEFT or RIGHT was displayed using their corresponding hand and they were instructed to make no hand movement when the word REST was displayed. The Philips IViewBOLD software was used to reveal significant regions of BOLD signal activation within left M1 (RIGHT > REST) and right M1 (LEFT > REST) in real-time. VOIs (20 × 20 × 20 mm) were centred at the peak of the fMRI BOLD activations localised within the left and right M1 regions. A further control VOI (V1) was placed using anatomical landmarks within the posterior region of the occipital lobe centred on the mid-sagittal plane.

MR spectroscopy data were collected sequentially from these three different brain regions using a Stimulated Echo Acquisition Mode (STEAM) sequence (TE/TM/TR = 16/17/2000 ms, BW = 4000 Hz, points = 4096, VOI = 80 ml). The three VOIs were scanned for each participant in a randomised order in the pre-tDCS period and were scanned in the reverse order in the post-tDCS period. 288 spectra were collected individually with Multiply Optimized Insensitive Suppression Train (MOIST) water suppression, and two spectra were acquired without water suppression for correction to absolute concentrations using water referencing. Each VOI scan took approximately 10 min to complete. Participants were asked to remain as still as possible during the scan.

### Data processing

Spectroscopy data were collected separately from each of the 32 coil elements. Data were realigned, phase corrected, and averaged, and then combined across coils with the ratio of signal to the square of the noise weighting (Hall et al., 2014) using a Matlab script (Mathworks inc. Natick, USA) developed in-house for this purpose. Spectra were then analysed using LCmodel software (version 2.2-4, Provencher, 1993) for quantification of major excitatory and inhibitory metabolites (i.e., GABA, Glutamate, and Glutamine). One participant in the cathodal group had to be excluded from the entire spectroscopy analysis due to a technical problem.

In all cases the shape of the spectrum was visually inspected and any data with an abnormal spectrum were excluded from further analysis. As a result, one participant in the sham group was excluded from the spectroscopic analysis of the left M1 VOI. Additionally, any outputs with a %SD greater than 60 in either pre-tDCS scan or post-tDCS scan were excluded from the analysis.

The concentrations of GABA and Glutamate were corrected for the proportion of grey matter within the voxel, and concentrations of NAA + NAAG were corrected for the proportion of the grey and white matter within the voxel, using the MPRAGE anatomical image (Stagg et al., 2009). Glutamine was not corrected as it is known to exist in significant concentrations within cerebrospinal fluid (Eckstein et al., 2008).

To verify that the VOIs (voxels) were equivalent, pre- and post-tDCS, a set of mixed ANOVAs were conducted to examine any changes in grey and white matter fractions within each VOI. The ANOVA consisted on a within-subject factor–time (pre- vs. post-tDCS) and a between-subject factor–tDCS polarity (anodal vs. cathodal vs. sham). The analyses confirmed that in all cases there were no significant main or interaction effects (all p > 0.05). These analyses confirm that tissue fractions pre- and post-tDCS did not differ. Furthermore, to further verify that small changes in VOI placement pre- and post-tDCS did not contribute to our findings, we also tested whether there was any association between pre vs. post-tissue fraction differences and GABA concentration change values. These analyses confirmed that there was no significant correlation between change in GABA concentration and change in tissue fraction.

In all cases neurochemical concentrations are given below as a ratio of simultaneously acquired NAA + NAAG concentrations after first verifying that there were no significant differences between in NAA + NAAG concentrations for the pre-tDCS session and post-tDCS session, or between any group (p > .05).

## Results

### Neurochemical changes induced by tDCS

Change ratios between the pre-tDCS and post-tDCS scans were calculated ([POST-PRE/PRE] × 100) separately for GABA, Glutamate, and Glutamine, for each VOI. Mean data for each group are shown in Table 1. Group comparisons were carried out using a priori planned contrasts in which the anodal and cathodal group data were each separately compared to the sham group using independent t-tests. These analyses revealed a significant difference between the anodal and sham groups for GABA change ratios (mean ± SD, anodal: − 19.77 ± 38.36%; sham: 35.06 ± 71.63%; t(13.77) = 2.13, p = 0.051, two-tailed, panel a). There was no significant difference in GABA ratio between the cathodal (cathodal mean: 17.78 ± 40.51%) and sham groups. Also there were no significant differences between any group for the Glutamate, and Glutamine change ratios (all p > .05).

**Table 1:** Neurochemical changes induced by anodal, cathodal, and sham tDCS.

	GABA		Glutamate		Glutamine	
	Subjects	Change ratio (%)	Subjects	Change ratio (%)	Subjects	Change ratio (%)
*Left M1*						
Anodal	10	− 19.77 ± 38.36	12	− 1.34 ± 14.36	11	2.73 ± 48.19
Cathodal	11	17.78 ± 40.51	12	− 4.58 ± 8.90	12	14.96 ± 53.87
Sham	10	35.06 ± 71.63	10	− 5.74 ± 15.28	10	− 1.26 ± 33.02

*Right M1*						
Anodal	9	22.16 ± 62.03	12	− 3.53 ± 10.72	12	− 15.73 ± 54.84
Cathodal	12	24.29 ± 68.53	12	− 5.42 ± 9.58	11	− 2.40 ± 57.77
Sham	10	23.46 ± 52.47	11	− 5.69 ± 7.70	11	− 7.87 ± 24.01

*V1*						
Anodal	11	59.51 ± 93.15	12	3.27 ± 9.36	12	5.83 ± 28.84
Cathodal	12	8.17 ± 69.37	12	− 2.46 ± 5.59	12	− 5.53 ± 14.38
Sham	11	39.37 ± 51.53	11	1.16 ± 12.72	11	2.23 ± 22.49

### Motor learning performance (force adaptation and de-adaptation task)

The mean perpendicular reaching error was calculated on each trial and the median error across trials was calculated for each individual for the adaptation phase and de-adaptation phase separately. In the adaptation phase, errors for catch trials were excluded and only trials were a force was applied were included in the analysis. Participants who exhibited an error greater than ± 2 SD from the entire group mean were excluded from the adaptation phase and the de-adaptation phase separately. As a result, one participant from each anodal group and cathodal group was excluded in the adaptation phase analysis, and one participant was excluded from each anodal group and sham group in the de-adaptation phase analysis. A one-way ANOVA revealed no significant differences between the three groups in either the adaptation phase (group 1 = 1.46 ± 0.38, group 2 = 1.37 ± 0.44, group 3 = 1.32 ± 0.43) or the de-adaptation phase (group 1 = − 0.82 ± 0.24, group 2 = − 0.68 ± 0.20, group 3 = − 0.88 ± 0.26) (all p > 0.05).

**Fig. 3:**
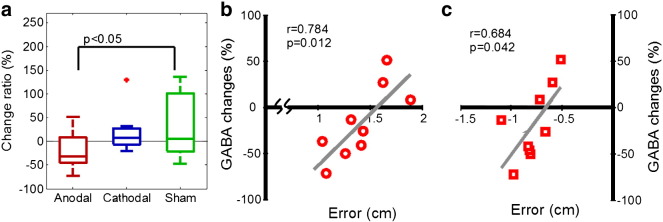
a. Left M1 GABA changes induced by anodal, cathodal, and sham tDCS. The box limits indicate the 25th and 75th percentiles, and the line inside the box shows the median. The whiskers indicate the range of the data within 1.5 times of the box width. b & c. Illustrate the relationship between MRS-GABA concentration change ratios induced by anodal tDCS and the movement error measured in the adaptation (b) and the de-adaptation phase (c) of the robot force adaptation task.

In the MR spectroscopy session, each group received either anodal, cathodal, or sham stimulation. Correlation analyses using Pearson's r were conducted to examine the relationship between tDCS-induced plasticity – measured by the change in GABA concentration change ratios induced by anodal, cathodal, or sham stimulation, and motor learning – measured by the reaching errors recorded during the adaptation and de-adaptation phases of the force adaptation task. These analyses revealed that there was a statistically significant correlation between individual motor learning performance in both the adaptation and the de-adaptation phases of the force adaptation task and GABA concentration change ratios measured within the left M1 VOI following anodal tDCS. These data are presented in Figs. 3c and d. The magnitude of the decrease in MRS-GABA in left M1 induced by anodal tDCS was positively associated with the magnitude of error during the adaptation phase (Pearson's r = 0.78, p < 0.05) and the de-adaptation phase (Pearson's r = 0.68, p < 0.05) of the force adaptation task. Participants who showed large decreases in GABA after anodal tDCS performed better (i.e., exhibited smaller errors during the adaptation phase) and increased motor memory retention (i.e., larger errors in the de-adaptation phase) of the force adaptation task. It should be noted however that, for the group receiving anodal tDCS only, force adaptation and de-adaptation performance were correlated with one another. A partial correlation analyses further revealed that while force de-adaptation performance remained strongly positively associated with GABA change ratios (r = 0.61) once force adaptation scores had been accounted for, this effect no longer reached conventional levels of statistical significance (p > 0.1).

There was no significant correlation observed between motor learning and MRS-GABA for either the group given cathodal tDCS or those given sham stimulation (both p > 0.05). Also, there was no significant correlations observed between motor learning measurements and Glutamate or Glutamine change ratios induced by tDCS, or between baseline (pre-tDCS) levels of GABA, Glutamate, or Glutamine (all p > 0.05).

## Discussion

In the current study we investigated how individual differences in motor learning and motor memory performance, measured using a robotic force adaptation and de-adaptation task, were predicted by tDCS-induced plastic changes in brain chemistry, in particular decreases in the inhibitory neurotransmitter GABA, measured using ultra-high-field (7 T) ^1^H magnetic resonance spectroscopy. The main results of this study are summarised below.

First, participants in each stimulation group showed clear evidence of learning and memory: as indexed by the magnitude of the mean errors observed in the force adaptation and de-adaptation phases of the robotic force perturbation task. It should be noted however that these group data also reflect inter-subject variability in the degree to which individual participants were able to learn and then un-learn the force perturbation applied during reaching by the robot.

Second, using ultra-high-field (7 T) ^1^H MRS we demonstrated that anodal tDCS delivered to the hand area of the left M1 induced a significant mean reduction in GABA concentration within the stimulated region. This tDCS-induced alteration in metabolite concentration was specific to GABA and was not observed for other key metabolites (e.g., Glutamate, Glutamine, and NAA). Importantly, this reduction was *polarity specific*, as it was not observed following either cathodal or sham stimulation, and also *spatially specific* in that no significant changes in GABA concentration were found within the contralateral (right) M1 region or in a control site located within V1.

Third, the magnitude of the tDCS-induced alterations in measured concentrations of GABA were shown to be highly correlated with both motor learning during the robotic force adaptation task and motor memory as indexed by performance during the force de-adaptation task. Specifically, we showed that individuals who exhibited large reductions in MRS-GABA concentration after anodal tDCS performed better on the force adaptation task (i.e., exhibited smaller errors during the adaptation phase) and exhibited increased motor memory retention (i.e., they produced larger errors in the force de-adaptation phase). These results are discussed below.

### Effects of offline anodal tDCS on MRS-GABA concentrations

tDCS can induce both online and after-effects on cortical excitability. The online effects of tDCS appear to be mainly due to alterations in membrane potential, whereas the after-effects of tDCS are thought to be dependent upon alterations in membrane potential and also changes in synaptic plasticity, particularly Glutamate and GABA signalling (for a review see Stagg and Nitsche (2011)). Both are likely to influence learning and memory. Our finding that offline anodal tDCS delivered to motor cortex leads to a significant decrease in MRS-GABA within the stimulated region, but not in two separate control sites, is consistent with a previous report (Stagg et al., 2009).

Our findings diverge however from those reported by Stagg et al. (2009) with respect to the effects of cathodal tDCS. While Stagg et al. (2009) reported that cathodal tDCS led to significant decreases in both Glutamate and GABA relative to sham stimulation, in our study we observed that there were no significant changes in either Glutamate or GABA after Cathodal tDCS in any of the regions (VOIs) measured. This discrepancy between the two studies may be due to several factors. First, there was difference in the intensity of the current used in these studies. Stagg and colleague applied 1 mA of cathodal tDCS for 10 min, whereas in the current study we applied 1.5 mA of cathodal tDCS for 15 min. The decision to use a slightly higher intensity within the present study was taken as a previous investigation had reported that 1 mA of tDCS was not sufficient to influence motor learning (Cuypers et al., 2013) and because it has been assumed that the duration and strength of tDCS after-effects increase linearly with an increase in the duration and the intensity of the applied stimulation (Nitsche and Paulus, 2001). However, a recent study by Batsikadze et al. (2013) has indicated that there may in fact be a non-linear relationship between cortical excitability changes induced by cathodal tDCS and current intensity. Specifically, they report that at higher current intensities, such as 2 mA, cathodal tDCS reverses its effect and leads to an increase in cortical excitability rather than a decrease.

Second, increases in current amplitude influence the timing of any effects observed for cathodal tDCS (Batsikadze et al., 2013). Specifically, while the effects of cathodal tDCS can be seen immediately after 1 mA stimulation, at 2 mA they are not observed until 90 min after stimulation. In the current study it is likely that post-stimulation MRS scanning was completed within 90 min of ceasing stimulation.

### tDCS-induced alterations in MRS-GABA predict motor learning and memory

As noted above, tDCS can induce both online and after-effects on cortical excitability. While the online effects of tDCS are thought to be due to alterations in membrane potential, the post-stimulation effects of tDCS appear to depend upon changes in synaptic plasticity, including changes in Glutamate and GABA signalling (Stagg and Nitsche, 2011).

Galea et al. (2010) have demonstrated that *online* anodal tDCS delivered to the cerebellum, but not the M1, led to significantly faster adaptation of reaching movements to a visuomotor transformation (a counter-clockwise rotation of visual feedback of hand position). By contrast, they showed that anodal tDCS applied to M1, but not the cerebellum, produced an increase in the retention of the learnt visuomotor transformation. They interpreted these findings as demonstrating that online anodal tDCS can enhance both motor learning and motor memory, and that while M1 may play a critical role in motor memory consolidation, the cerebellum may play more an important role for motor learning.

The force adaptation task used in the current study is conceptually similar to the visuomotor adaptation task reported by Galea et al. (2010). Participants in the current study learnt to adapt to a counter-clockwise force perturbation delivered by a robotic manipulandum during reaching movements and, after learning had taken place, to de-adapt to the removal of the force perturbation. The key finding is that tDCS-induced plasticity, indexed by alterations in MRS-GABA, is a significant predictor of motor learning and memory, as measured by the magnitude of errors made during adaptation and de-adaptation on the robotic force perturbation task. Specifically individuals with larger tDCS-induced decreases in MRS-GABA exhibited reduced reaching errors during adaptation (learning) and increased reaching errors during de-adaptation (memory). Importantly, this finding extends the work reported by Galea et al. (2010) by demonstrating: that offline tDCS. i.e., the after-effects of tDCS stimulation, to M1 may influence both learning and memory performance; that the offline effect of anodal tDCS is to decrease MRS-GABA concentrations; and, that individual differences in tDCS-induced alterations in MRS-GABA predict the efficacy of motor learning and motor memory.

It is of interest to note that in the current study, whereas individual differences in baseline GABA concentration (i.e., MRS-GABA measured prior to tDCS being delivered) did not predict individual motor learning performance, measured either during the adaptation or de-adaptation phases of the force perturbation study. Instead it was the tDCS-induced change in MRS-GABA that predicted motor learning. This lack of any significant correlation between baseline MRS-GABA level and motor learning performance is consistent with the findings reported by Stagg et al. (2011) who found that baseline MRS-GABA levels were positively correlated with measures of performance (e.g. mean reaction time) but did not predict learning performance in a serial reaction time paradigm. By contrast, Stagg et al. (2011) reported a positive correlation between the degree of GABA responsiveness to anodal tDCS and individual differences in learning (i.e., measured as reductions in mean RT) on sequence learning task which involved learning a repeating sequence of manual button press responses.

It has been argued previously that the force adaptation task used in the present study may involve a different form of learning to that observed in the serial reaction time task (Krakauer and Mazzoni, 2011). Specifically, while the force perturbation task is thought to involve adaptation to a predicable force through the formation of an internal dynamic ‘forward’ model, the sequence learning task is thought to be a form of a skill learning that is more dependent on success-based state-space exploration (i.e., habit or model-free learning). Importantly, the results of the current study, together with those reported by Stagg et al. (2011), indicate that tDCS-induced plasticity in primary motor cortex – as indexed by alterations in MRS-GABA following anodal stimulation – predicted motor learning performance for both tasks. This indicates that GABAergic activity within the primary motor cortex may play an important role in modulating cortical excitability thus influencing in motor learning and memory across a wide range of tasks.
